# 

**Published:** 1883-10

**Authors:** 


OCTOBER, 1883.

### THE AMERICAN JOURNAL OF DENTAL SCIENCE.

LIBRARY
INDEXED
S.G.O.

PUBLISHED MONTHLY.

$2.50 PER ANNUM.

EDITED BY

F. J. S. GORGAS, M. D., D. D. S.

VOL. 17. THIRD SERIES. NO. 6.

BALTIMORE:
SNOWDEN & COWMAN.

LONDON:
TRUBNER & CO., 60 PATERNOSTER ROW.

1883.

